# Response rates and representativeness from birth to young adulthood in a Swedish birth cohort

**DOI:** 10.1093/ije/dyag196

**Published:** 2026-09-11

**Authors:** Niklas Andersson, Anna Zettergren, Maria Ödling, Elin Dahlén, Göran Pershagen, Magnus Wickman, Anne-Sophie Merritt, Erik Melén, Inger Kull, Sandra Ekström, Anna Bergström

**Affiliations:** Institute of Environmental Medicine, Karolinska Institutet, Stockholm, Sweden; Institute of Environmental Medicine, Karolinska Institutet, Stockholm, Sweden; Department of Clinical Science and Education, Södersjukhuset, Karolinska Institutet, Stockholm, Sweden; Department of Women’s and Children’s Health, Karolinska Institutet, Stockholm, Sweden; Department of Clinical Science and Education, Södersjukhuset, Karolinska Institutet, Stockholm, Sweden; Network for Pharmacoepidemiology, The NEPI Foundation, Stockholm, Sweden; Institute of Environmental Medicine, Karolinska Institutet, Stockholm, Sweden; Institute of Environmental Medicine, Karolinska Institutet, Stockholm, Sweden; Institute of Environmental Medicine, Karolinska Institutet, Stockholm, Sweden; Centre for Occupational and Environmental Medicine, Region Stockholm, Stockholm, Sweden; Department of Clinical Science and Education, Södersjukhuset, Karolinska Institutet, Stockholm, Sweden; Sachs’ Children’s and Youth Hospital, Södersjukhuset, Stockholm, Sweden; Department of Clinical Science and Education, Södersjukhuset, Karolinska Institutet, Stockholm, Sweden; Sachs’ Children’s and Youth Hospital, Södersjukhuset, Stockholm, Sweden; Institute of Environmental Medicine, Karolinska Institutet, Stockholm, Sweden; Department of Clinical Science and Education, Södersjukhuset, Karolinska Institutet, Stockholm, Sweden; Centre for Occupational and Environmental Medicine, Region Stockholm, Stockholm, Sweden; Institute of Environmental Medicine, Karolinska Institutet, Stockholm, Sweden; Centre for Occupational and Environmental Medicine, Region Stockholm, Stockholm, Sweden

**Keywords:** response rates, representativeness, asthma, birth cohort, epidemiology

## Abstract

**Background:**

In birth-cohort studies, response rates generally decline over time. We aimed to evaluate representativeness and identify predictors of retention up to young adulthood in a population-based birth cohort originally focused on asthma and allergies.

**Methods:**

The Barn/Child, Allergy, Milieu, Stockholm, Epidemiology (BAMSE) birth cohort was established in 1994–6 in Stockholm, Sweden. We utilized data from questionnaires and Swedish registers to compare the characteristics of participants at age 24 years (*n* = 3064) with those of the original cohort (*n* = 4089) and the general population born in 1994–6 in Stockholm county (*n* = 68 378) and Sweden (*n* = 310 976). Logistic regression was used to identify predictors of retention at age 24 years.

**Results:**

The response rate declined over time and was higher in females than in males in adolescence and young adulthood (80.0% versus 70.0% at age 24 years). Compared with the original cohort, participants at age 24 years more often had dispensed asthma medications (6.6% versus 5.8%) and university education (52.6% versus 47.8%), but had less often a mother who smoked during pregnancy (9.6% versus 10.8%). Similar patterns were observed in comparisons with the general population. Female sex [odds ratio (OR) 1.75, 95% confidence interval (CI) 1.49–2.05] and high parental education (OR 1.33, 95% CI 1.12–1.59) were the predominant predictors of retention in the cohort.

**Conclusion:**

Female sex, higher socio-economic status, and asthma were predictive factors of retention in the cohort after 24 years. This may have influenced the observed prevalence rates of asthma and allergies, and associations between potential risk factors and outcomes associated with retention.

Key Messages

- We aimed to evaluate representativeness and identify predictors of retention up to young adulthood in a population-based birth cohort originally focused on asthma and allergies.
- Females, participants with higher socio-economic status, and those with asthma were more likely to remain in the cohort after 24 years.
- The differences in retention may have influenced the observed prevalence rates of asthma and allergies, and associations between potential risk factors and outcomes associated with retention.

## Introduction

Birth-cohort studies provide a unique possibility to evaluate the incidence, prevalence, and factors associated with common disorders such as allergic and respiratory diseases [1]. Continued follow-up of established birth cohorts is crucial for understanding the onset and progression of disease and identifying targets for disease prevention and mitigation. More than 100 birth cohorts focusing on asthma and allergy have been initiated around the world and several are being performed in the general population [2, 3]. However, only a few have followed participants up to adulthood [4–8].

Participation in longitudinal studies is often described by the response rate, attrition, and/or retention [9]. While the response rate refers to participation at a given time point, attrition describes the cumulative loss of participants over time and retention the proportion of the original cohort that remains engaged. In general, response rates in epidemiological studies have declined over the past decades [10, 11]. Moreover, the response rate is generally lower in some subgroups, e.g. males [9, 10], individuals with lower socio-economic position [9, 10], unemployed people [10], and ethnic minorities [9]. In longitudinal studies, response rates often decline for each successive follow-up [11, 12], affecting both attrition and retention. However, the way in which the decline in retention rates influences the representativeness in birth-cohort studies with long follow-up remains unclear [2, 11].

With >24 years of follow-up and access to Swedish population-based registers, the BAMSE (Barn/Child, Allergy, Milieu, Stockholm, Epidemiology) birth cohort provides an excellent setting in which to evaluate representativeness and identify predictors of retention. Thus, the aim of the present study was to examine the representativeness of the participants in the 24-year follow-up compared to the original cohort as well as the general population of Sweden. We also aimed to identify predictors of retention in the cohort.

## Methods

### BAMSE birth cohort

The BAMSE cohort is a population-based birth cohort with the original aim of assessing risk factors for asthma and other allergic diseases in childhood and factors of importance for the prognosis of already established allergic disease [13]. In 1994–6, infants born in predefined areas of Stockholm, Sweden were selected for the study. Of 7221 newborn children, 5488 were eligible and 4089 families responded to the baseline questionnaire and were included in the study after some exclusion criteria, e.g. the family planned to move within 1 year and insufficient knowledge of the Swedish language [13]. The mean age at inclusion was 2.1 months.

The participants were followed up at ages of around 1, 2, 4, 8, 12, 16, and 24 years by using questionnaires sent to the parents (up to age 16 years) and to themselves (from age 12 years). At each follow-up, all individuals who had not permanently withdrawn (*n* = 289) or died (*n* = 12) were invited, regardless of participation in the latest wave. At the 4-, 8-, 16-, and 24-year follow-ups, all participants who responded to the questionnaire were invited for a clinical examination. Multiple retention strategies (e.g. careful design of questionnaires, repeated reminders, study webpage, and webinars) have been used to keep a high response rate. More information on the study design and retention strategies can be found in the Supplementary files, including the timeline (Supplementary Figure 1) and flowchart (Supplementary Figure 2) for the BAMSE cohort.

### General population of Stockholm county and Sweden

For the general-population groups, individuals born in 1994–6 and living in Stockholm county and Sweden, respectively, were chosen. The years of birth were chosen to capture a population of the same age as the participants in the BAMSE birth cohort. The groups were identified by Statistics Sweden. Data from the general population were obtained for the first year of life (Stockholm county: *n* = 68 378, the whole of Sweden: *n* = 310 976) and for the year in which each respective individual turned 23 years old (Stockholm county: *n* = 82 124, the whole of Sweden: *n* = 376 100), i.e. in 2017, 2018, or 2019, to overlap with the 24-year follow-up (November 2016 to May 2019). For the adult population, all participants fulfilling the criteria, regardless of country of birth, were included, in contrast to the birth-year population, which only included individuals born in Stockholm county and Sweden, respectively.

### Registries

For the comparisons between the cohort and the general population, aggregated data from the registries of Statistics Sweden and the National Board of Health and Welfare in Sweden were collected through linkage with personal identity numbers [14]. Data from the Total Population Register (containing data on population composition) [15], the Educational Attainment of the Population Register (containing data on education) [16], and the Income and Tax Statistics Register (containing data on salaries) were used. The National Medical Birth Register (containing data on pregnancies, labour, and newborns) [17] and the National Prescribed Drug Register (NPDR, containing data on dispensed drug prescription) [18] were also used.

For income variables, the mean exchange rates between the Swedish krona (SEK) and the Euro (EUR) were used for 2017, 2018, and 2019 (for details, see Supplementary files).

Information from the NPDR on dispensed medications was classified in accordance with the Anatomical Therapeutic Chemical (ATC) Classification System [19] (any dispensed medication excluding birth-control pills, first ATC level of D for dermatological medications, and R03 for asthma medications).

### Statistical analysis

Descriptive data are presented in percentages or specified units. Relative percentage differences are presented as the absolute percentage change divided by the reference value. Comparisons of response rates between females and males were performed by using two-tailed *t*-tests. Logistic regression was used to investigate associations between early-life characteristics as reported in the questionnaires and responding to the 24-year questionnaire. Analyses were performed with Stata (version 19.5; StataCorp LLC, 2025, College Station, TX, USA).

## Results

### Response rates and cohort characteristics

**Figure 1:**
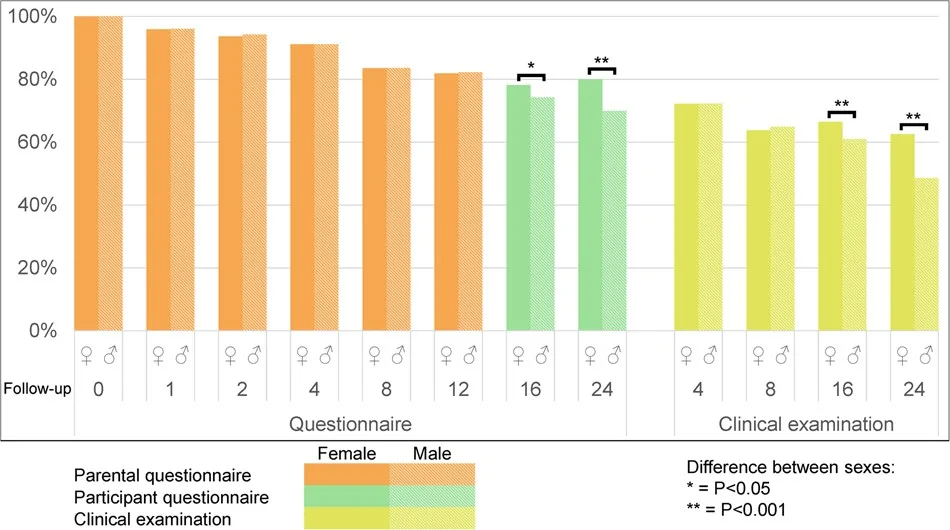
Response and participation rates in the BAMSE birth cohort. ♀ = female, ♂ = male, and the number refer to the respective year of follow-up.

At baseline, 4089 children participated in the cohort (2024 females and 2065 males). The response rates to the questionnaires and clinical examinations up to age 24 years are shown in Fig. 1. The response rate declined for each follow-up. At age 24 years, 74.9% (*n* = 3064) responded to the questionnaire and 55.5% (*n* = 2270) participated in the clinical examination. At participant ages 16 and 24 years, the response rates for both the questionnaire and the clinical examination were higher in females than in males [80.0% in females versus 70.0% in males for the questionnaire at age 24 years, absolute difference 10.0%, 95% confidence interval (CI) 7.4–12.7].

### Representativeness of the cohort

Table 1 presents the distribution of selected demographic characteristics from the national Swedish registers among the participants in the 24-year follow-up and the original cohort and in the general populations of Sweden and Stockholm, respectively. The relative differences between the 24-year questionnaire responders and the original cohort and the general population are illustrated in Supplementary Figures 3 and 4, respectively.

**Table 1:** Prevalence rates for variables from different registries in the population responding to the 24-year questionnaire, participating in the clinical examination at the 24-year follow-up, the BAMSE original cohort, and the general populations of Stockholm county and Sweden. All populations were born in 1994, 1995, or 1996. For the general populations (Sweden and Stockholm county), the data are for everyone born in those years, for the first year of life (baseline) and the year they turned 23 years old, respectively.

Variable	Questionnaire at 24 years	Clinical examination at 24 years	BAMSE original cohort	General population of	
				Stockholm county	Sweden
**Baseline year**					
Number of individuals	3064	2270	4089	68 378	310 976
Female	52.8%	55.7%	49.4%	48.5%	48.8%
Married mother (including registered partner)	50.9%	51.0%	50.0%	51.6%	47.2%
Mother’s age ≤25 years at child’s birth	7.0%	6.6%	7.6%	14.0%	17.4%
At least one parent with university education	63.5%	64.4%	60.9%	48.8%	40.2%
At least one parent born outside Sweden	21.4%	21.4%	22.6%	32.4%	23.0%
Living in a household with at least four people	40.4%	39.6%	41.8%	52.6%	57.3%
Maternal underweight (BMI < 18.5 kg/m^2^) in early pregnancy^a^^,b^	3.5%	3.7%	3.5%	3.7%	3.1%
Maternal overweight or obesity (BMI > 25 kg/m^2^) in early pregnancy^a^^,b^	20.1%	18.9%	20.3%	24.5%	29.2%
Smoking mother (at least one cigarette/day) in early pregnancy^a^^,c^	9.6%	8.8%	10.8%	15.0%	17.2%
Pregnancy length (<37 weeks)^a^	5.2%	5.2%	5.3%	5.8%	6.0%
Pregnancy length (≥42 weeks)^a^	9.1%	9.4%	9.0%	7.6%	6.9%
Delivered by caesarean section^a^	12.6%	12.7%	12.7%	13.5%	12.1%
Estimated median disposable yearly income for the household (EUR^d^) after inflation^e^	35 290	35 402	34 797	31 873	30 340
**Year of twenty-third birthday**					
Number of individuals	3064	2270	4089	82 124	376 100
Any university education^f^	52.6%	55.5%	47.8%	36.9%	33.7%
Employed	64.0%	65.3%	64.2%	67.0%	65.9%
Living with at least one parent	45.9%	46.7%	47.9%	44.7%	33.7%
Born in Sweden and at least one parent born outside Sweden	21.0%	21.4%	21.5%	24.4%	17.3%
Born outside Sweden	0%	0%	0%	24.7%	20.3%
Living in a household with at least four people	26.3%	26.5%	27.3%	35.3%	26.8%
Estimated median disposable yearly income for the household (EUR^d^)	50 637	52 071	51 805	54 656	44 065
Estimated median disposable yearly income (EUR^d^)	11 097	11 131	11 835	14 523	15 397
Any dispensed medications (excluding birth-control pills)	54.8%	57.9%	52.3%	48.6%	47.2%
Dispensed dermatological medications (D first ATC level)	12.7%	13.3%	12.3%	11.2%	9.3%
Dispensed asthma medications (ATC: R03)	6.6%	7.4%	5.8%	4.5%	4.6%

a For Stockholm county and Sweden, the prevalence is based on 66 571 and 305 793 individuals, respectively.

b Missing data for between 14% and 18% of all mothers on BMI.

c Missing data for between 6.4% and 9.2% of all mothers on smoking.

d Exchange rate of 1 Euro (EUR) = 10.1608 Swedish Krona (SEK), mean exchange rate between 1 January 2017 and 31 December 2019.

e Mean yearly inflation of 31.923% in Sweden between 1994–2017, 1995–2018, and 1996–2019.

f Missing data for between 1.4% and 5.8% of the individuals.

Otherwise, missing between 0 and 3.4%.

BMI, body mass index.

For the baseline variables, the largest relative differences between the responders to the 24-year questionnaire and the original cohort were observed for maternal smoking in early pregnancy (9.6% versus 10.8%, relative difference −11.2%), young maternal age (7.0% versus 7.6%, relative difference −7.7%), and female sex (52.8% versus 49.4%, relative difference +6.9%) (Table 1 and Supplementary Figure 3). For the variables collected in adulthood, the largest relative differences were observed for dispensed asthma medications (6.6% versus 5.8%, relative difference +14.0%), having any university education (52.6% versus 47.8%, relative difference +10.0%), and estimated median disposable yearly income (11 097€ versus 11 835€, relative difference −6.2%) (Table 1 and Supplementary Figure 3). Small differences were seen for the other investigated variables, e.g. pregnancy-related factors (Supplementary Figure 3). Similar patterns, but with somewhat larger differences, were observed when participants attending the clinical follow-up were compared to the original cohort (Supplementary Figure 3). Moreover, stratified analyses showed similar patterns among males and females, although with somewhat larger differences for males (Supplementary Tables 2 and 3).

When the 24-year questionnaire responders were compared with the general population of Stockholm county, the largest relative differences for the baseline variables were observed for having a young mother at birth (7.0% versus 14.0%, relative difference −50.1%), maternal smoking in early pregnancy (9.6% versus 15.0%, relative difference −36.3%), and having at least one parent born outside Sweden (21.4% versus 32.4%, relative difference −34.0%) (Table 1 and Supplementary Figure 4). For the same comparison for variables collected in adulthood, the largest differences were observed for having dispensed asthma medications (6.6% versus 4.5%, relative difference +46.3%), having any university education (52.6% versus 36.9%, relative difference +42.4%), and living in a household of at least four people (26.3% versus 35.3%, relative difference −25.6%) (Table 1 and Supplementary Figure 4).

Comparisons between the 24-year questionnaire responders and the general population of Sweden showed similar patterns to the comparisons with the general population of Stockholm county (Supplementary Figure 3) except that having a parent born outside Sweden was more common among the 24-year questionnaire responders compared with the general population of Sweden (21.0% versus 17.3%, relative difference +21.8%). By design, all participants in the BAMSE birth cohort were born in Sweden, whereas 24.7% of the population of Stockholm county and 20.3% of the general population of Sweden were born outside Sweden during the years 2017–19 (Table 1 and Supplementary Figure 4). When the 24-year questionnaire responders were compared to the general population excluding immigrants (individuals born outside Sweden), the proportions with at least one parent born outside Sweden (21.0%) was somewhat lower than in Stockholm county (30.5%), but similar to Sweden (21.6%). For other variables, excluding immigrants had limited influence (data not shown).

### Prediction of participation in the 24-year follow-up

Table 2 presents the distribution of selected baseline characteristics collected at enrolment into the cohort among the questionnaire responders and participants in the clinical examination at the 24-year follow-up and in the original cohort. Similarly to when register variables were used, there were higher proportions of females responding to the 24-year questionnaire (52.8% versus 49.5%) and participants from households with higher socio-economic status (white-collar parents) (84.5% versus 82.7%) and/or with at least one parent with university/college education (55.5% versus 52.9%) compared with those in the original cohort. For other characteristics (e.g. parental country of birth, heredity of allergic disease, parental smoking, birth municipality, air-pollution levels during the first year of life, wheeze, and eczema during the first 2 years of life), there were no or small differences. Similar differences from the original cohort were seen for participants in the clinical examination at age 24 years.

**Table 2:** Description of the populations of the BAMSE original cohort, those responding to the 24-year questionnaire, and those participating in the clinical examination at the 24-year follow-up.

Variable	BAMSE original cohort	Questionnaire at 24 years	Clinical examination at 24 years
	n = 4089	n = 3064	n = 2270
Female	49.5	52.8	55.8
Older siblings	48.4	46.8	46.2
White-collar parent	82.7	84.5	84.8
At least one parent with university feducation	52.9	55.5	56.9
Mother’s age ≤25 years at child’s birth	7.8	7.2	6.9
At least one parent born outside Sweden^a^	21.1	20.7	20.9
Parental heredity of allergic disease	29.7	30.5	31.2
Maternal smoking during pregnancy or infancy	13.8	12.8	12.3
Parental smoking during infancy	21.0	20.4	20.3
Furred pet at baseline	15.4	15.3	14.6
Exclusively breast fed for ≥4 months	79.5	80.4	80.2
Mould exposure at baseline	25.3	25.5	25.0
Birthweight (mean gram)	3530	3531	3523
Birth length^b^ (mean mm)	501.8	501.7	501.3
Pregnancy length^c^ (25–36 weeks)	5.4	5.3	5.4
Pregnancy length^c^ (37–41 weeks)	85.5	85.5	85.1
Pregnancy length^c^ (42–44 weeks)	9.1	9.2	9.5
Birth onset^d^—spontaneous	87.0	87.0	86.9
Birth onset^d^—with induction	8.4	8.4	8.4
Birth onset^d^—caesarean section	4.6	4.7	4.7
NO_*x*_ during first year (µg/m^3^) [mean (SD)]	31.9 (17.9)	32.3 (18.2)	32.8 (18.5)
PM_10_ during first year (µg/m^3^) [mean (SD)]	14.8 (2.5)	14.9 (2.6)	14.9 (2.6)
Soot during first year (µg/m^3^) [mean (SD)]	1.04 (0.45)	1.05 (0.46)	1.06 (0.46)
PM_2.5_ during first year (µg/m^3^) [mean (SD)]	8.9 (0.97)	8.9 (0.98)	9.0 (0.99)
Birth municipality—Stockholm	29.6	31.1	32.6
Birth municipality—Järfälla	28.8	28.3	27.6
Birth municipality—Solna	26.4	25.9	25.9
Birth municipality—Sundbyberg	15.2	14.5	14.0
Any wheeze during the first 2 years^e^	26.1	25.9	26.6
Any eczema during the first 2 years^f^	25.8	26.1	26.7

a Based on responses at 8 years: 16.6% missing for BAMSE original cohort, 11.6% missing for the questionnaire at 24 years, and 10.2% missing for the clinical examination at 24 years.

b 13.6% missing for BAMSE original cohort, 9.8% missing for the questionnaire at 24 years, and 8.5% missing for the clinical examination at 24 years.

c Data from birth registry if missing from 4-year questionnaire.

d Data from birth registry.

e Based on responses up to 2 years: 6.7% missing for BAMSE original cohort, 4.6% missing for the questionnaire at 24 years, and 3.9% missing for the clinical examination at 24 years.

f Based on responses up to 2 years: 6.6% missing for BAMSE original cohort, 4.4% missing for the questionnaire at 24 years, and 3.9% missing for the clinical examination at 24 years.

NO_*x*_, nitrogen oxides (nitric oxide, NO, and nitrogen dioxide, NO_2_).

PM_10_, particulate matter with a diameter of ≤10 micrometres; PM_2.5_, particulate matter with a diameter of ≤2.5 micrometres.

In multiple logistic regression analyses (Table 3), the odds of responding to the 24-year questionnaire were higher for females [odds ratio (OR) 1.75, 95% CI 1.49–2.05], those with at least one parent with any university education (OR 1.33, 95% CI 1.12–1.59), and those with a furred pet at baseline (OR 1.27, 95% CI 1.02–1.59). Moreover, there was an inverse association between maternal smoking during pregnancy or infancy (OR 0.79, 95% CI 0.63–0.99) and responding to the 24-year questionnaire.

**Table 3:** Associations between early-life characteristics and response to the 24-year questionnaire—crude and mutually adjusted logistic regression.

Variable	Odds ratio (95% CI)	
	Crude	Mutually adjusted
	*N* = 4089	*N* = 3668
Female	**1.72 (1.49–1.98)**	**1.75 (1.49–2.05)**
Mother’s age ≤25 years at child’s birth	**0.72 (0.56–0.93)**	0.97 (0.72**–**1.31)
At least one parent with university education	**1.51 (1.31–1.74)**	**1.33 (1.12–1.59)**
Parental heredity of allergic disease	**1.18 (1.00–1.38)**	1.17 (0.98–1.39)
White-collar parent	**1.59 (1.33–1.90)**	1.23 (0.99–1.53)
Exclusively breastfed for ≥4 months	**1.24 (1.04–1.48)**	1.15 (0.95–1.39)
Furred pet at baseline	0.99 (0.81–1.20)	**1.27 (1.02–1.59)**
Maternal smoking during pregnancy or infancy	**0.72 (0.60–0.88)**	**0.79 (0.63–0.99)**
Birth municipality—Stockholm	Ref.	Ref.
Birth municipality—Järfälla	**0.73 (0.61–0.89)**	0.85 (0.68–1.05)
Birth municipality—Solna	**0.73 (0.60–0.89)**	0.81 (0.66–1.01)
Birth municipality—Sundbyberg	**0.65 (0.52–0.81)**	0.83 (0.64–1.07)
Any wheeze during the first 2 years^a^	0.94 (0.80–1.12)	1.05 (0.87–1.26)
Any eczema during the first 2 years^b^	1.05 (0.89–1.25)	1.06 (0.88–1.27)

a Based on questions at 1 and 2 years with 6.7% missing.

b Based on questions at 1 and 2 years with 6.6% missing.

Estimates and CIs in bold when *P* < .05.

## Discussion

This study investigated how a population-based birth cohort developed over more than two decades of follow-up. Overall, after 24 years, 74.9% of the participants remained. The response rate declined over time and more females than males stayed in the cohort. The remaining participants also had indications of higher socio-economic status (e.g. higher degree of university education) compared with those in the original cohort. Similar but larger differences were observed when the cohort participants were compared with the same-aged general population. Additionally, in multivariable analyses, female sex and high parental education were predictive of retention in the cohort at age 24 years.

In line with our findings, a decline in response rates over time has also been observed in birth cohorts in Europe and Australia [7, 11, 20–26]. Among birth cohorts with long follow-up, the German MAS cohort included 71.6% [27, 28] and the Australian Raine cohort included 51.0% of the original cohort at age 20 years [7], while the British ALSPAC cohort included 50% at age 18 years [8]. Thus, the response rate in our cohort was somewhat higher. In contrast to our findings, no difference in response rates between females and males was observed in the MAS cohort [27] and sex-specific response rates were not reported in Raine or ALSPAC [7, 28]. However, higher response rates among females have previously been reported in Swedish epidemiological studies [29, 30] and a review of birth cohorts of very preterm infants showed a higher loss to follow-up among males [11].

Although our cohort maintained a high retention rate over time, national register data indicated that participants who remained after 24 years tended to have higher socio-economic status, e.g. shown by higher parental education. This is in line with findings from other birth-cohort studies in Europe and Australia [7, 11, 20–26]. Moreover, compared with the same-aged general populations of Stockholm county, having a parent born outside of Sweden was less common among BAMSE participants. Sensitivity analyses excluding individuals born outside Sweden from the general population reduced these differences. The differences regarding socio-economic factors and country of birth may be partly explained by the predefined recruitment areas for the BAMSE birth cohort, as well as the inclusion criterion of having good knowledge of the Swedish language [13]. In addition, BAMSE is a closed cohort that includes only participants born in Stockholm county, whereas the same-aged general population of Stockholm includes individuals who have immigrated to Sweden.

The somewhat lower prevalence of maternal smoking during early pregnancy among the participants remaining in the cohort is in line with findings from other birth cohorts [20, 21] and also aligns with our previous finding that parental smoking was significantly more prevalent in families not responding to the baseline questionnaire [13]. It is possible that other lifestyle factors are also associated with the willingness to participate and remain in cohort studies.

In analyses based on individual participant data, female sex and high parental education were identified as the most important predictors of retention in the cohort when using data from the baseline questionnaire. This is in line with our findings based on national registers, but has the advantage of allowing multivariable analyses, which is important, as many of the studied factors are correlated. Similar predictors of retention have also been reported in a few birth cohorts, including the 1958 British National Child Development Study, in which female sex predicted retention in the follow-ups at ages of ≥23 years, but not at younger ages [29, 31].

Another important finding in our study is the somewhat higher proportion of individuals with asthma medication compared with the original cohort as well as the general population, indicating a higher retention in participants with asthma. Although differential loss to follow-up has been recognized as a limitation of birth-cohort studies [3], few studies have been able to assess this potential bias. In contrast to our findings, a study from the Danish national birth cohort showed a somewhat lower prevalence of asthma among the participants included in the 7-year follow-up compared with those not included [21]. However, the BAMSE birth cohort was initially designed to study allergic and respiratory diseases, reflected by a focus on symptoms of asthma, rhinitis, eczema, and food allergy in the questionnaires and clinical examinations [32, 33]. Therefore, individuals with asthma and allergies may have found the study more relevant, which could have resulted in a higher retention rate within that group. Despite this, the prevalence of asthma in our cohort is comparable to that in Swedish national surveys [34] and other epidemiological studies [6, 35–37], indicating that this is not a major source of bias in our study. Similarly, the prevalence rates of other allergic diseases such as rhinitis and eczema in our cohort [38] have been shown to be comparable to those in other studies [37] and national surveys [34]. Moreover, in line with this, previous analyses in the Danish national birth cohort have shown that the observed associations between exposure and outcome were not biased by non-participants [39]. Still, if the loss to follow-up is related to the exposure and outcome of interest, then the validity of the observed associations may be violated. Unfortunately, we were not able to explore this potential bias in our study, as we did not have access to individual data from the registers. For the same reason, we could not use register data to identify predictors of retention in the cohort. Additionally, as sex-specific analyses can provide information on potential biological mechanisms, a lower response rate in one sex may lead to lack of power in such analyses. Therefore, monitoring the follow-up rates in different groups can be an important strategy in longitudinal studies that continue over many years. Specific retention strategies may be needed to improve the response rate in specific groups, e.g. males. Alternatively, the potential influence of non-random loss to follow-up may be handled by applying survey weights in the analyses of the data (i.e. to give higher weights to participants from groups with lower response rates) [40]. In BAMSE, we have not yet used survey weights in the analyses.

A major strength of this study is the possibility to compare the prevalence of a number of factors among participants who remained after 24 years to both the original cohort and the general population by using registry data with high reliability and coverage [41–44]. However, the use of aggregated register data limited our possibilities to perform detailed analyses. A strength of the BAMSE cohort is the complementary retention strategies, including reminders, barrier reductions, and bond building, resulting in low attrition throughout the years.

In summary, after 24 years of follow-up of the BAMSE birth cohort, we had high retention, with 74.9% answering the questionnaire. Females and participants with higher socio-economic status were more likely to remain in the cohort, whereas other socio-demographic factors showed no or relatively small differences compared with the original cohort and the general population of the same age. Participants with asthma were somewhat more likely to remain in the cohort. This may have influenced the observed prevalence of asthma and allergies as well as the validity of the observed associations between potential risk factors and outcomes, if these are associated with cohort retention. Moreover, our findings highlight the importance of monitoring the non-response and actively working with retention strategies to minimize the loss to follow-up in birth-cohort studies.

## Ethics approval

The BAMSE study was approved by the Regional Ethics Committee at Karolinska Institutet in Stockholm, Sweden.

## Supplementary Material

dyag196_Supplementary_Data

## Data Availability

The data underlying this article cannot be shared publicly due to the privacy of individuals who participated in the study.
