# Supplementary material for: Response rates and representativeness from birth to young adulthood in a Swedish birth cohort
Source: Int J Epidemiol. 2026 Sep 11;55(5):dyag196. doi: 10.1093/ije/dyag196 (PMC13568472; doi:10.1093/ije/dyag196)
Supplement: Supplementary file 1 [file dyag196_supplementary_data.zip › Supplementary/ije-2026-01-0086-File004_new.docx]

Supplementary file:

# Response rate and representativeness from birth to young adulthood in a Swedish birth cohort

Niklas Andersson, Anna Zettergren, Maria Ödling, Elin Dahlén, Göran Pershagen, Magnus Wickman, Anne-Sophie Merritt, Erik Melén, Inger Kull, Sandra Ekström and Anna Bergström

## The BAMSE birth cohort

The BAMSE (Barn/Child, Allergy, Milieu, Stockholm, Epidemiology) birth cohort was established in Stockholm, Sweden 1994-1996. Families with a newborn child living in four pre-defined areas of Stockholm and planning to reside in their home for at least one year were eligible for the study. The geographic areas were selected to cover a variation in socioeconomic status and environmental factors including different housing settings, e.g., suburbs and urban and inner-city parts of Stockholm (Järfälla, Sundbyberg, Solna, and parts of Stockholm municipality). Furthermore, the families had to have sufficient knowledge of Swedish, and those with a seriously ill child were not eligible. A close collaboration was established with the 58 children's health centres located in the selected study areas, and the eligible families received the first information about the study through their nurse at the child healthcare centre. In most cases, the first (baseline) questionnaire was distributed through the health care centre, while some families received it by mail. Recruitment started in February 1994 and to reach sufficient power to answer the original research questions, recruitment continued until at least 4000 children were included, which occurred in November 1996. In total, 7221 children were born in the recruitment area during this period. Of these, 477 could never be reached and 1256 were actively excluded based on the following exclusion criteria: the family planned to move within one year, the parents had insufficient knowledge of Swedish, the child were seriously, the family already had a child included in the study. Taken together, 5488 children were eligible, and 4089 were included in the study.

The baseline questionnaire included questions on heredity of allergic disease, environmental and lifestyle factors. Follow-up questionnaires eliciting information on allergic symptoms, doctor diagnosis and medication use as well as selected environmental and lifestyle exposures were sent when the children were one, two, four, eight, 12, 16 and 24 years. Up to the eight-year follow-up, postal questionnaires were sent out. At the 12-year follow-up (2008), web-based questionnaires were introduced. These were answered by almost 69% of the responders, while 23% answered a postal questionnaire and 8% answered via phone. At the 16-year follow-up, 91% responded the web-based questionnaire and 9% a postal questionnaire. At the 24-year follow-up, only a few participants answered a postal questionnaire, while the majority answered the web-based version. The questionnaires had between 62 and 171 questions (including several gatekeeper questions, reducing the number of questions for many participants). At the follow-ups at 16 and 24 years, the questionnaires were estimated to take around 20 up to 30 minutes. From infancy to age 16 years, the invitations and questionnaires were sent to the parents. At ages 12 and 16 years, parents and children answered questionnaires with complementary questions. At age 24 years, the invitation and questionnaire were sent to the participants themselves, while the parents did not participate. However, the parents received information about the upcoming study and a summary of key findings from the BAMSE birth cohort.

At ages four, eight, 16 and 24 years, all participants who had answered the questionnaire were invited to a clinical examination including assessment of height, weight and lung function as well as blood sampling for analyses of e.g. immunoglobin E (IgE) levels. Each clinical examination was designed to take approximately one hour.

Throughout the follow-ups, great efforts have been made to keep a high response-rate to the questionnaires and clinical examinations. The questionnaires have been carefully designed to be easy to answer, and the feasibility of the questionnaire has been tested before each wave. Advance letters, announcing the upcoming study have been used in some of the follow-ups. After distribution of the questionnaire, repeated reminders were sent by post or e-mail and thereafter non-responders were contacted by telephone. At ages 12 and 24 years, a possibility to respond to core-questions by telephone was used. This was done by 15% (n=493) of the participants at age 12 years and 5% (n=154) at age 24 years. Additionally, at ages 16 and 24 years, a gift card of around 50€ (500 SEK) was given to participants who responded to the questionnaire and participated in the clinical examinations. At all clinical follow-ups, we have aimed to have flexible booking systems and opening hours, to facilitate participation. After the clinical examinations, participants (or their parents up to age 16 years), received written information on the results of their test of allergen-specific immunoglobin E antibodies (IgE-sensitisation). Between the waves of follow-up, we have been working with bond-building strategies, e.g. with the study website[1], seminars, workshops and dissemination of results from the study in Swedish media.

In figures S1 and S2 the timeline and flowchart of the BAMSE cohort is presented.

**Figure S1** The timeline of the BAMSE (Barn/Child, Allergy, Milieu, Stockholm, Epidemiology) cohort showing the timing of the different follow-ups.

**Figure S2** BAMSE (Barn/Child, Allergy, Milieu, Stockholm, Epidemiology) flowchart. ♀ = Female, ♂ = Male

## Description of baseline characteristics

The definitions of the baseline characteristics are shown in table S1. The air pollution variable (NO_x_, PM_10_, Soot and PM_2.5_) was assessed using dispersion modelling[2].

**Table S1** List of definitions of baseline characteristics

| **Variable** | **Definition** |
| --- | --- |
| Mothers age at birth | Younger (≤25 years) |
|  | Older (>25 years) |
|  |  |
| Parental education | Higher (at least one parent with university/collage education) |
|  | Lower (no parent with university/collage education) |
|  |  |
| Parental heredity | Yes (at least one parent having a doctor's diagnosis of asthma in combination with asthma medication, doctor's diagnosis of hay fever in combination with furred pet or pollen allergy |
|  | No (no parent having a doctor's diagnosis of asthma in combination with asthma medication, doctor's diagnosis of hay fever in combination with furred pet or pollen allergy |
|  |  |
| Socioeconomic status for the household | White collar (at least one parent with a white-collar profession) |
|  | Blue collar (both parents with blue collar profession |
|  |  |
| Furred pet exposure | Yes (cat, dog or rodent at home) |
|  | No (no cat, dog or rodent at home) |
|  |  |
| Maternal smoking | Yes (mother smoked at least one cigarette per day at baseline or any point of time during the pregnancy) |
|  | No (mother not smoking at least one cigarette per day at baseline or any point of time during the pregnancy) |
|  |  |
| Municipality | Stockholm |
|  | Järfälla |
|  | Solna |
|  | Sundbyberg |
|  |  |
| Mould exposure | Yes (smell of mildew, visible mould/mildew or moister damage) |
|  | No (no smell of mildew, visible mould/mildew or moister damage) |
|  |  |
| Exclusive breast fed^a^ | Yes (exclusive breast fed for at least four months) |
|  | No (exclusive breast fed for less than four months) |
|  |  |
| Wheeze during the two first years of life^b^ | Yes (at least one episode of wheeze after the age of three months and up to the date of questionnaire at two years, i.e. during the two first years of life) |
|  | No (no episode of wheeze after the age of three months and up to the date of questionnaire at two years) |
|  |  |
| Eczema during the two first years of life^b^ | Yes (dry skin in combination with itchy rash for two weeks or more and typical localisation [face, arms/legs extension surfaces, arms/legs flexures or wrists/ankles flexures] or doctor's diagnosis of eczema) |
|  | No (no dry skin or itchy rash) |
|  |  |
| Parental ethnicity^c^ | Outside of Sweden (at least one parent born outside of Sweden) |
|  | In Sweden (both parents born in Sweden) |
|  |  |
| ^a^From the questionnaire at one year | |
| ^b^From the questionnaire at one and two years | |
| ^c^From the questionnaire at eight years | |

## Description of register variables

Information on country of birth was obtained from the Total Population Register[3]; country of birth was categorised as Sweden vs. outside of Sweden.

Information on maternal BMI was obtained from the National Medical Birth Register[4] containing information height and weight recorded at the enrolment visit at the maternity care centre. BMI was calculated as body weight in kilograms divided by height in meters squared (kg/m^2^) and classified according to the World Health Organization's recommendations: underweight (< 18.5 kg/m^2^), normal weight (18.5–24.9 kg/m^2^) and overweight or obesity (≥ 25 kg/m^2^).

Information on maternal smoking (yes vs. no) was obtained from the National Medical Birth Register[4].

## Exchange between SEK to EUR

For all income variables the mean exchange rate between the Swedish krona (SEK) and the Euro (EUR) during 1^st^ of January 2017 and 31^st^ of December 2019 were used, 1 EUR = 10.1608 SEK[5]. To account for inflation between the earlier years we used the mean yearly inflation between 1994-2017 (32.5%), 1995-2018 (32.1%) and 1996-2019 (31.2%)[6] which was 31.9%.

**Figure S3** Heatmap with relative comparisons (% difference) between BAMSE (Barn/Child, Allergy, Milieu, Stockholm, Epidemiology) original cohort and the populations that responded to the 24-year questionnaire and participated in the clinical examination at the 24-year follow-up, respectively.

**Figure S4** Heatmap with relative comparisons (% difference) between the general population of Stockholm county and Sweden and the populations that responded to the 24-year questionnaire, participated in the clinical examination at the 24-year follow-up, and the BAMSE (Barn/Child, Allergy, Milieu, Stockholm, Epidemiology) original cohort. All participants are born 1994, 1995, or 1996; for the general population (Stockholm county and Sweden), data on everyone born in those years are included for the first year of life and the year they turned 23 years old, respectively.

**Table S2** Female rates for variables from different registries in the population responding to the 24-year questionnaire, participating in the clinical examination at the 24-year follow-up, the BAMSE (Barn/Child, Allergy, Milieu, Stockholm, Epidemiology) original cohort, and the general populations of Stockholm county and Sweden. All populations are born 1994, 1995, or 1996. For the general populations (Sweden and Stockholm county), the data are for everyone born in those years, for the first year of life (baseline) and the year they turned 23 years old, respectively.

| **Variable** | **Questionnaire at 24 years** | **Clinical examination at 24 years** | **BAMSE original cohort** | **General population of** | |
| --- | --- | --- | --- | --- | --- |
|  |  |  |  | **Stockholm** **county** | **Sweden** |
| **BASELINE YEAR** |  |  |  |  |  |
| Number of individuals | 1619 | 1266 | 2024 | 33 161 | 151 773 |
| Married mother (including registered partner) | 51.8% | 51.6% | 51.6% | 47.3% | 51.9% |
| Mother’s age at child’s birth ≤25 years | 7.2% | 6.7% | 7.8% | 17.4% | 13.9% |
| At least one parent with university education | 61.8% | 61.9% | 60.1% | 39.5% | 47.8% |
| At least one parent born outside Sweden | 22.6% | 23.0% | 23.2% | 22.9% | 32.2% |
| Living in a household with at least four people | 40.3% | 40.2% | 41.2% | 57.3% | 52.5% |
| Maternal underweight (BMI <18.5 kg/m^2^) in early pregnancy^a, b^ | 3.8% | 3.8% | 3.7% | 3.2% | 3.7% |
| Maternal overweight or obesity (BMI >25 kg/m^2^) in early pregnancy^a, b^ | 19.6% | 18.0% | 19.6% | 29.2% | 24.6% |
| Smoking mother (≥1 cigarette/day) in early pregnancy^a, c^ | 9.6% | 9.0% | 10.4% | 17.2% | 14.7% |
| Pregnancy length <37 weeks^a^ | 5.1% | 5.3% | 5.2% | 5.7% | 5.5% |
| Pregnancy length ≥42 weeks^a^ | 7.3% | 7.8% | 7.2% | 6.2% | 6.8% |
| Delivered by caesarean section^a^ | 11.6% | 12.0% | 11.8% | 11.6% | 12.8% |
| Estimated median disposable yearly income for the household (EUR^d^) after inflation^e^ | 33 838 | 33 801 | 33 351 | 29 111 | 30 500 |
| **YEAR OF 23^RD^ BIRTHDAY** |  |  |  |  |  |
| Number of individuals | 1619 | 1266 | 2024 | 40 381 | 181 534 |
| Any university education^f^ | 56.9% | 58.6% | 53.6% | 40.3% | 42.8% |
| Gainfully employed | 64.7% | 66.6% | 64.2% | 64.7% | 67.2% |
| Living with at least one parent | 41.4% | 42.7% | 43.7% | 28.1% | 39.0% |
| Born in Sweden and at least one parent born outside Sweden | 22.4% | 23.1% | 22.4% | 17.4% | 24.4% |
| Born outside Sweden | 0% | 0% | 0% | 19.6% | 24.2% |
| Living in a household with at least four people | 23.9% | 24.0% | 25.1% | 25.4% | 33.4% |
| Estimated median disposable yearly income for the household (EUR^d^) | 47 642 | 48 987 | 48 418 | 41 410 | 50 717 |
| Estimated median disposable yearly income (EUR^d^) | 10 352 | 10 542 | 10 501 | 12 663 | 12 853 |
| Any dispensed medications (excluding birth control pills) | 64.4% | 66.2% | 62.7% | 57.7% | 58.6% |
| Dispensed dermatological medications (D first ATC level) | 15.0% | 14.8% | 15.0% | 11.8% | 13.9% |
| Dispensed asthma medications (ATC: R03) | 7.6% | 8.2% | 6.9% | 5.4% | 5.4% |
| ^a^For Stockholm county and Sweden, the prevalence is based on 32 315 and 149 238 individuals respectively. | | | | | |
| ^b^Missing data for between 14–18% of all mothers on BMI. | | | | | |
| ^c^Missing data for between 7.3–9.0% of all mothers on smoking. | | | | | |
| ^d^Exchange rate of 1 Euro (EUR) = 10.1608 Swedish Krona (SEK), mean exchange rate between 1st of January 2017 to 31st of December 2019. | | | | | |
| ^e^Mean yearly inflation in Sweden between 1994–2017, 1995–2018, and 1996–2019. | | | | | |
| ^f^Missing data for between 1.9–5.5% of the individuals. | | | | | |
| Otherwise, missing data for between 0–4.0%. | | | | | |
| BMI, Body Mass Index | | | | | |
| ATC, Anatomical Therapeutic Chemical | | | | | |

**Table S3** Male rates for variables from different registries in the population responding to the 24-year questionnaire, participating in the clinical examination at the 24-year follow-up, the BAMSE (Barn/Child, Allergy, Milieu, Stockholm, Epidemiology) original cohort, and the general populations of Stockholm county and Sweden. All populations are born 1994, 1995, or 1996. For the general populations (Sweden and Stockholm county), the data are for everyone born in those years, for the first year of life (baseline) and the year they turned 23 years old, respectively.

| **Variable** | **Questionnaire at 24 years** | **Clinical examination at 24 years** | **BAMSE original cohort** | **General population of** | |
| --- | --- | --- | --- | --- | --- |
|  |  |  |  | **Stockholm** **county** | **Sweden** |
| **BASELINE YEAR** |  |  |  |  |  |
| Number of individuals | 1445 | 1004 | 2065 | 35 217 | 159 203 |
| Married mother (including registered partner) | 49,9% | 50,2% | 48,4% | 47,2% | 51,3% |
| Mother’s age at child’s birth ≤25 years | 6,7% | 6,5% | 7,3% | 17,4% | 14,1% |
| At least one parent with university education | 65,3% | 67,3% | 61,4% | 39,6% | 48,1% |
| At least one parent born outside Sweden | 20,0% | 19,3% | 21,9% | 22,9% | 32,2% |
| Living in a household with at least four people | 40,5% | 38,8% | 42,3% | 57,3% | 52,6% |
| Maternal underweight (BMI <18.5 kg/m^2^) in early pregnancy^a, b^ | 3,2% | 3,5% | 3,4% | 3,0% | 3,7% |
| Maternal overweight or obesity (BMI >25 kg/m^2^) in early pregnancy^a, b^ | 20,5% | 20,0% | 21,0% | 29,3% | 24,4% |
| Smoking mother (≥1 cig/day) in early pregnancy^a, c^ | 9,5% | 8,5% | 11,1% | 17,2% | 15,3% |
| Pregnancy length <37 weeks^a^ | 5,2% | 5,1% | 5,4% | 6,3% | 6,1% |
| Pregnancy length ≥42 weeks^a^ | 11,1% | 11,2% | 10,8% | 7,6% | 8,3% |
| Delivered by caesarean section^a^ | 13,7% | 13,6% | 13,6% | 12,6% | 14,1% |
| Estimated median disposable yearly income for the household (EUR^d^) after inflation^e^ | 33 689 | 33 994 | 33 313 | 29 111 | 30 558 |
| **YEAR OF 23^RD^ BIRTHDAY** |  |  |  |  |  |
| Number of individuals | 1445 | 1004 | 2065 | 41 743 | 194 566 |
| Any university education^f^ | 47,8% | 51,6% | 42,2% | 27,4% | 31,3% |
| Gainfully employed | 63,2% | 63,8% | 64,2% | 67,0% | 66,8% |
| Living with at least one parent | 50,9% | 51,7% | 52,0% | 39,0% | 50,2% |
| Born in Sweden and at least one parent born outside Sweden | 19,5% | 19,2% | 20,7% | 17,1% | 24,4% |
| Born outside Sweden | 0% | 0% | 0% | 21,0% | 25,2% |
| Living in a household with at least four people | 28,9% | 29,5% | 29,5% | 28,2% | 37,3% |
| Estimated median disposable yearly income for the household (EUR^d^) | 50 320 | 52 126 | 51 142 | 43 046 | 54 166 |
| Estimated median disposable yearly income (EUR^d^) | 11 295 | 10 957 | 12 266 | 17 482 | 15 219 |
| Any dispensed medications (excluding birth control pills) | 44,1% | 47,5% | 42,2% | 37,4% | 38,8% |
| Dispensed dermatological medications (D first ATC level) | 10,2% | 11,4% | 9,6% | 7,0% | 8,6% |
| Dispensed asthma medications (ATC: R03) | 5,5% | 6,3% | 4,7% | 3,7% | 3,7% |
| ^a^For Stockholm county and Sweden, the prevalence is based on 34 256 and 156 555 individuals respectively. | | | | | |
| ^b^Missing data for between 13–18% of all mothers on BMI. | | | | | |
| ^c^Missing data for between 5.3–9.3% of all mothers on smoking. | | | | | |
| ^d^Exchange rate of 1 Euro (EUR) = 10.1608 Swedish Krona (SEK), mean exchange rate between 1st of January 2017 to 31st of December 2019. | | | | | |
| ^e^Mean yearly inflation in Sweden between 1994–2017, 1995–2018, and 1996–2019. | | | | | |
| ^f^Missing data for between 0.8–6.1% of the individuals. | | | | | |
| Otherwise, missing data for between 0–2.9% | | | | | |
| BMI, Body Mass Index | | | | | |
| ATC, Anatomical Therapeutic Chemical | | | | | |

# References

1.	Karolinska Institutet. *BAMSE Projektet.* https://ki.se/bamse (14 Jul. 2026, date last accessed).

2.	Segersson D, Eneroth K, Gidhagen L *et al.* Health impact of PM10, PM2.5 and black carbon exposure due to different source sectors in Stockholm, Gothenburg and Umea, Sweden. *Int J Environ Res Public Health* 2017;**14**(7). https://doi.org/10.3390/IJERPH14070742,.

3.	Statistics Sweden (Statistikmyndigheten SCB). *Mikrodata för Registret över totalbefolkningen (RTB).* https://www.scb.se/vara-tjanster/bestall-data-och-statistik/register/registret-over-totalbefolkningen-rtb/ (30 Oct. 2024, date last accessed).

4.	The National Board of Health and Welfare (Socialstyrelsen). *National Medical Birth Register.* https://www.socialstyrelsen.se/en/statistics-and-data/registers/national-medical-birth-register/ (30 Oct. 2024, date last accessed).

5.	The European Central Bank (ECB). *Swedish krona (SEK).* https://www.ecb.europa.eu/stats/policy_and_exchange_rates/euro_reference_exchange_rates/html/eurofxref-graph-sek.en.html (18 Sep. 2024, date last accessed).

6.	Statistics Sweden (Statistiska Centralbyrån). *Prisomräknaren.* https://www.scb.se/hitta-statistik/sverige-i-siffror/prisomraknaren/ (18 Sep. 2024, date last accessed).
